# Varenicline versus transdermal nicotine patch for smoking cessation: results from a randomised open-label trial

**DOI:** 10.1136/thx.2007.090647

**Published:** 2008-07-28

**Authors:** H-J Aubin, A Bobak, J R Britton, C Oncken, C B Billing, J Gong, K E Williams, K R Reeves

**Affiliations:** 1 Hôpital Emile Roux, Assistance Publique-Hopitaux de Paris, Limeil-Brévannes; Centre d’Enseignement, de Recherche et de Traitement des Addictions, Hôpital Paul Brousse, Paris; Assistance Publique-Hôpitaux de Paris; INSERM, U669, Paris, France; 2 Wandsworth Medical Centre, London, UK; 3 University of Nottingham, Nottingham, UK; 4 University of Connecticut Health Center, Farmington, Connecticut, USA; 5 Pfizer Global Research and Development, Groton, Connecticut, USA

## Abstract

**Background:**

Varenicline, a new treatment for smoking cessation, has demonstrated significantly greater efficacy over placebo and sustained release bupropion (bupropion SR). A study was undertaken to compare a 12-week standard regimen of varenicline with a 10-week standard regimen of transdermal nicotine replacement therapy (NRT) for smoking cessation.

**Methods:**

In this 52-week, open-label, randomised, multicentre, phase 3 trial conducted in Belgium, France, the Netherlands, UK and USA, participants were randomly assigned (1:1) to receive varenicline uptitrated to 1 mg twice daily for 12 weeks or transdermal NRT (21 mg/day reducing to 7 mg/day) for 10 weeks. Non-treatment follow-up continued to week 52. The primary outcome was the biochemically confirmed (exhaled carbon monoxide ⩽10 ppm) self-reported continuous abstinence rate (CAR) for the last 4 weeks of the treatment period in participants who had taken at least one dose of treatment. Secondary outcomes included CAR from the last 4 weeks of treatment through weeks 24 and 52, and measures of craving, withdrawal and smoking satisfaction.

**Results:**

A total of 376 and 370 participants assigned to varenicline and NRT, respectively, were eligible for analysis. The CAR for the last 4 weeks of treatment was significantly greater for varenicline (55.9%) than NRT (43.2%; OR 1.70, 95% CI 1.26 to 2.28, p<0.001). The week 52 CAR (NRT, weeks 8–52; varenicline, weeks 9–52) was 26.1% for varenicline and 20.3% for NRT (OR 1.40, 95% CI 0.99 to 1.99, p = 0.056). Varenicline significantly reduced craving (p<0.001), withdrawal symptoms (p<0.001) and smoking satisfaction (p<0.001) compared with NRT. The most frequent adverse event was nausea (varenicline, 37.2%; NRT, 9.7%).

**Conclusions:**

The outcomes of this trial established that abstinence from smoking was greater and craving, withdrawal symptoms and smoking satisfaction were less at the end of treatment with varenicline than with transdermal NRT.

**Trial registration number:**

NCT00143325.

Smoking cessation treatments are among the most cost-effective disease prevention interventions available.^1^ Many smokers want to quit smoking, but unaided quit attempts have 1-year success rates of only 3–5%.^2^ ^3^ Pharmacological treatments approved for smoking cessation in the USA and the European Union include various forms of nicotine replacement therapy (NRT), sustained-release (SR) bupropion and, most recently, varenicline.^1^ ^4^ ^5^ Compared with placebo, NRT and bupropion SR approximately double the odds of remaining abstinent 6–12 months after quitting,^6^ ^7^ while varenicline raises the odds by 2.5–3 times compared with placebo 12 months after quitting.^8^^–^10

Bupropion SR and NRT are both recognised as first-line pharmacotherapies for smoking cessation in the US and Europe,^1^ ^4^ and the most commonly used in the UK and the USA is NRT.^11^ ^12^ Direct comparison of varenicline with bupropion SR in two double-blind clinical trials showed that varenicline had significantly greater efficacy than bupropion SR at the end of 12 weeks of treatment and at 6 months of follow-up,^8^ ^10^ and one of these studies showed continued significant efficacy at 1-year follow-up.^10^ Smoking cessation treatment with varenicline also resulted in greater verified abstinence 4 weeks after quitting than single-use NRT treatment in a historical comparison of consecutive routine cases before and after the introduction of varenicline.^13^

We report the results of the first randomised clinical trial comparing varenicline with transdermal nicotine. The primary objective of the present study was to compare a 12-week standard regimen of varenicline with a 10-week standard regimen of transdermal NRT for smoking cessation using an open-label design.

## METHODS

### Study design

This was an open-label randomised trial conducted in 24 centres in Belgium (4 sites), France (6 sites), The Netherlands (4 sites), UK (4 sites) and USA (6 sites). Ethical approval was gained from Independent Review Boards for each centre. The study, conducted from 17 January 2005 to 28 June 2006, complied with the ethical principles of the Declaration of Helsinki and the International Conference on Harmonization Good Clinical Practices Guidelines. All participants provided written informed consent prior to any procedures.

### Study population

Participants were all motivated to stop smoking and were recruited in smoking cessation clinics or via local advertising. They were smokers, 18–75 years of age, weight >45.5 kg and body mass index 15–38 kg/m^2^. Each participant smoked at least 15 cigarettes per day with no period of abstinence >3 months in the previous year. Female smokers were eligible providing they were not breastfeeding, pregnant or at risk of becoming pregnant. Participants were excluded if they had a history of cancer, any other serious or unstable disease within the previous 6 months, diagnoses of or treatment for depression or other psychological disorder, or drug or alcohol dependence within the previous 12 months. Other exclusion criteria were clinically significant allergic reactions to drugs or adhesive tapes, skin disorders, systolic blood pressure >150 mm Hg or diastolic blood pressure >95 mm Hg, clinically significant renal or hepatic impairment, evidence of liver dysfunction or other abnormal laboratory tests. Participants were also excluded if they were taking medication that may interfere with the study outcome, had previously participated in a varenicline study in the previous year or had used of any form of NRT in the previous 6 months.

### Interventions

After screening, eligible participants were invited to a baseline visit. Using a central computer-generated sequence, they were randomised in a 1:1 ratio to either 12 weeks of treatment with varenicline or 10 weeks of treatment with a nicotine transdermal patch (as per the manufacturer’s recommendation).^14^ ^15^ Participants were provided with an educational booklet on smoking cessation entitled “*Clearing the air: how to quit smoking … and quit for keeps*” at the baseline visit and took part in a brief (⩽10 min) counselling session in accordance with the US Public Health Service Guidelines.^1^ Counselling also occurred during every subsequent telephone and clinic visit.

**Figure 1:**
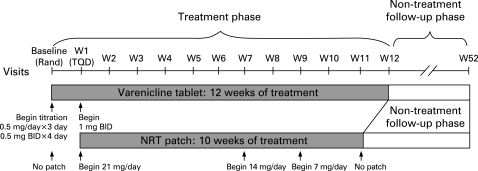
Study design. W, week; TQD, target quit date; Rand, randomisation; NRT, nicotine replacement therapy; BID, twice daily.

The target quit date (TQD) coincided with the week 1 visit in both treatment groups. Varenicline treatment began 1 week before the TQD (the day after the baseline visit) whereas NRT treatment began on the TQD as per the manufacturer’s recommendations. Participants randomised to varenicline were administered 0.5 mg/day for 3 days, 0.5 mg twice daily for 4 days, then 1 mg twice daily thereafter. Full dosing was achieved by the TQD and continued for a further 11 weeks. Participants randomised to NRT applied transdermal patches (NicoDerm CQ Clear (GlaxoSmithKline) in the USA and NiQuitin CQ Clear (GlaxoSmithKline) in Europe) each morning starting on the TQD for 10 weeks. Doses of NRT were 21 mg/day for the first 6 weeks, 14 mg/day for 2 weeks, then 7 mg/day for 2 weeks (as per the manufacturer’s recommendation). During the treatment phase, participants received a contact telephone call 3 days after the TQD and attended the clinic on a weekly basis commencing in week 1 (fig 1).

The non-treatment follow-up phase of the study totalled 41 weeks for NRT participants and 40 weeks for varenicline participants. Visits to the clinic (weeks 13, 16, 24, 32, 40, 48 and 52) were interspersed with telephone calls (weeks 14, 20, 28, 36 and 44).

### Study end points

#### Efficacy

The primary end point was the self-reported continuous abstinence rate (CAR), confirmed by exhaled carbon monoxide (CO) levels of 10 ppm or below, during the last 4 weeks of treatment (varenicline, weeks 9–12; NRT, weeks 8–11).

Secondary efficacy end points included CO-confirmed CAR for the last 4 weeks of treatment through weeks 24 and 52 (varenicline, weeks 9–24 and 9–52; NRT, weeks 8–24 and 8–52). Abstinence from smoking during the preceding 7 days and confirmed by CO measures (7-day point prevalence of abstinence) was also assessed at the end of treatment and at the week 24 and week 52 visits.

#### Measures of craving and withdrawal

The Minnesota Nicotine Withdrawal Scale (MNWS)^16^ ^17^ assessed urge to smoke, depressed mood, irritability, anxiety, poor concentration, restlessness, increased appetite and insomnia. The modified Cigarette Evaluation Questionnaire (mCEQ)^18^ ^19^ recorded measures of smoking satisfaction, psychological reward, enjoyment of respiratory tract sensations, craving reduction and aversion.

Both these questionnaires were completed at the baseline visit and at each weekly visit through week 7, or at termination for participants who discontinued the study before week 7. While the MNWS was completed by all participants, the mCEQ was only completed by participants who reported smoking since their last completed questionnaire. The MNWS was also completed on TQD+3.

### Safety evaluations

Blood chemistry, haematology, urinalysis tests, vital signs, physical examinations, body weight measures and electrocardiograms were assessed during the treatment period. All observed or self-reported adverse events were recorded and followed up until resolution or the end of study. Any adverse event that was life-threatening or resulted in hospitalisation, persistent or significant disability, incapacity or death was considered a serious adverse event, as was a medical or surgical intervention to prevent one of these outcomes.

### Statistical analysis

A sample size of 365 participants per group was estimated to provide at least 90% power to detect a difference between varenicline and NRT for the last 4 weeks of treatment CAR based on an odds ratio (OR) of 1.75 (assuming NRT cessation rate of 24%) and for the week 52 CAR based on an OR of 2.00 (assuming NRT cessation rate of 11.5%).

Measures of abstinence were treated as binary data and analysed using a logistic regression model, including terms for treatment and centre. Participants were classified as smokers or non-smokers for each end point and analyses were of abstinence rates. To be classified as a non-smoker, participants had to have not used any nicotine-containing product (other than a transdermal patch in the NRT group) during the treatment phase or any tobacco products during the follow-up phase. Use of NRT during the 9 months of follow-up did not disqualify a subject from being a responder provided other conditions were met. Participants who missed a visit but had otherwise met the criteria since the last visit were considered non-smokers. Missing CO data were assumed to be ⩽10 ppm provided other conditions were met. Participants who withdrew from the study were assumed to be smokers for the remainder of the study, regardless of their smoking status at the last visit.

Efficacy and safety analyses were conducted on randomised participants who received at least one dose of study medication (Primary Analysis Population). As a prespecified sensitivity analysis, identical analysis of CAR values was also conducted using the All Randomised Population, which additionally included any randomised participants who withdrew before receiving study drug. Owing to the 1-week difference in treatment duration, additional prespecified sensitivity analyses were conducted with 4-week CAR values for weeks 8–11, 9–12 and with week 52 CAR starting from week 8 and week 9 for both treatment groups.

The ORs and 95% confidence intervals (CIs) are estimates from the logistic regression model of CAR. The likelihood ratio χ^2^ test was used and significance tests were two-tailed, α = 0.05. The additional effect of treatment-by-centre interaction was determined from an expanded logistic model. A post hoc assessment of the effect of country was conducted using an expanded logistic model including treatment, country and treatment-by-country interaction.

Subscales of the MNWS and the mCEQ were analysed as continuous variables from the TQD through week 7. The analyses were based on a repeated measures model with treatment, baseline measure, centre, visit and treatment by visit interaction as factors. Model estimates on the average effect and p values were obtained by contrasting the average scores over week 1 through week 7. No imputation for missing subscale scores at individual visits was conducted. SAS V.8.2 (SAS Institute, Cary, North Carolina, USA) was used for all analyses.

## RESULTS

### Participants

**Figure 2:**
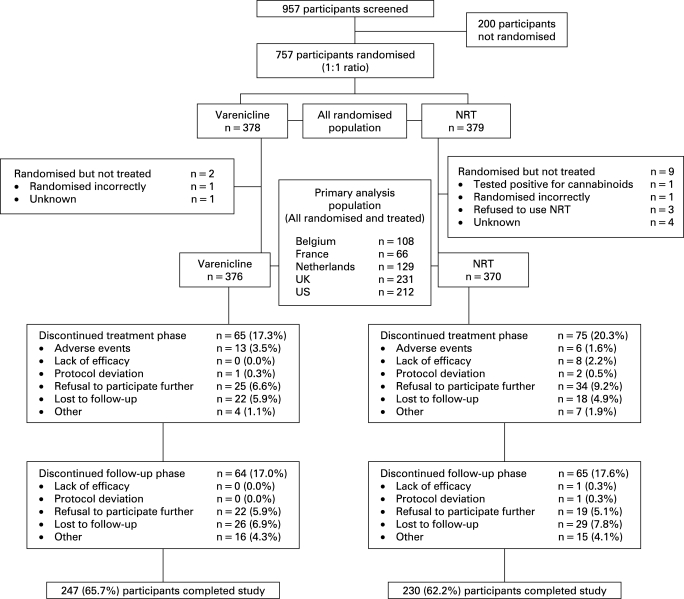
Participant disposition. NRT, nicotine replacement therapy.

A total of 957 participants were screened for the study and 757 were randomised (378 varenicline; 379 NRT). Of these, 376 varenicline participants and 370 NRT participants reported taking at least one dose of treatment medication. The progression of participants through the study is shown in fig 2. The demographic characteristics and smoking histories were comparable between treatment groups (table 1).

**Table 1:** Demographic characteristics and smoking history

Parameter	Varenicline(n = 376)	NRT(n = 370)
Mean (SD) age (years)	42.9 (10.5)	42.9 (12.0)
Mean (SD) height (cm)	171.9 (9.6)	171.6 (9.2)
Mean (SD) weight (kg)	76.4 (15.9)	75.6 (14.1)
Gender (%)		
Male	48.4	50.0
Female	51.6	50.0
Race (%)		
Caucasian	92.6	93.5
Black	2.9	1.6
Asian	2.1	2.4
Other	2.4	2.4
Mean (range) duration of smoking (years)	25.9 (2–58)	25.2 (1–62)
Mean (range) cigarettes per day over last month (n)	23.0 (15–80)	22.4 (11–60)
1 or more previous serious quit attempts (%)	86.1	89.7
Previous attempts with nicotine patch (%)	48.5	46.2
Previous attempts with bupropion (%)	19.7	20.0
Mean (range) longest previous period of abstinence (days)	5.64 (0–90)	7.49 (0–90)
Mean (SD) Fagerström test for nicotine dependence score	5.62 (2.23)	5.37 (1.99)

### Efficacy

#### End of treatment 4-week CAR

**Figure 3:**
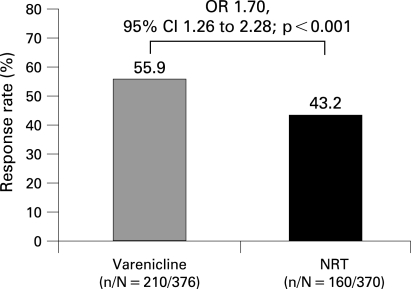
Four-week end of treatment CAR (weeks 9–12 for varenicline and weeks 8–11 for NRT) for the Primary Analysis Population (all randomised and treated participants who took at least one dose of study medication). Results of the All Randomised Population CAR for the same period: varenicline, 55.6%; NRT, 42.2% (OR 1.76, 95% CI 1.31 to 2.36; p<0.001). CAR, continuous abstinence rate; NRT, nicotine replacement therapy; OR, odds ratio; CI, confidence intervals.

For the Primary Analysis Population (all randomised and treated), the proportion of participants who remained continuously abstinent for the last 4 weeks of treatment was significantly greater for varenicline (weeks 9–12, 55.9% (n = 210)) than for NRT (weeks 8–11, 43.2% (n = 160); OR 1.70, 95% CI 1.26 to 2.28, p<0.001; fig 3). The results were similar for the All Randomised Population (varenicline, 55.6%; NRT, 42.2%; OR 1.76, 95% CI 1.31 to 2.36, p<0.001) and were also similar when the two treatments were compared in the same 4-week periods: weeks 9–12 (varenicline, 55.9%; NRT, 42.2%; OR 1.78, 95% CI 1.32 to 2.39, p<0.001) and weeks 8–11 (varenicline, 55.1%; NRT, 43.2%; OR 1.64, 95% CI 1.22 to 2.21, p<0.001).

#### CAR through week 24

In the Primary Analysis Population, week 24 CAR (NRT, weeks 8–24; varenicline, weeks 9–24) was higher for varenicline (32.4%) than for NRT (27.3%) but the difference was not significant (OR 1.29, 95% CI 0.94 to 1.77, p = 0.118). The results were similar in the All Randomised Population (varenicline, 32.2%; NRT, 26.6%; OR 1.33, 95% CI 0.97 to 1.82, p = 0.081).

#### CAR through week 52

**Figure 4:**
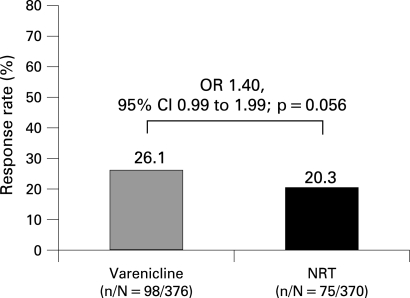
CAR through week 52 (weeks 9–52 for varenicline and weeks 8–52 for NRT) for the Primary Analysis Population (all randomised and treated participants who took at least one dose of study medication). Results of the All Randomised Population CAR for the same period: varenicline, 25.9%; NRT, 19.8% (OR 1.44, 95% CI 1.02 to 2.03; p = 0.040). CAR, continuous abstinence rate; NRT, nicotine replacement therapy; OR, odds ratio; CI, confidence intervals.

In the Primary Analysis Population, week 52 CAR (NRT, weeks 8–52; varenicline, weeks 9–52) was also higher for varenicline (26.1%; n = 98) than for NRT (20.3%; n = 75; OR 1.40, 95% CI 0.99 to 1.99, p = 0.056; fig 4). The results were similar in the All Randomised Population but the difference at week 52 was significant (varenicline, 25.9%; NRT, 19.8%; OR 1.44, 95% CI 1.02 to 2.03, p = 0.040).

#### CAR by study centre and country

There was no significant treatment-by-centre interaction for the 4-week end of treatment CAR or for week 52 CAR (NRT, weeks 8–52; varenicline, weeks 9–52), supporting the generalisation of combined results across centres in which this study was conducted. Post hoc analyses showed evidence of a treatment-by-country interaction for the end of treatment CAR (p = 0.009, table 2) but not through week 52 (p = 0.306). Additional post hoc analyses were performed to investigate the treatment-by-country interaction. There were no significant interactions for treatment-by-baseline characteristics (all nominal p values were >0.10 for the following categories: Fagerström test for nicotine dependence score, smoking rate, previous quit attempt with NRT or with transdermal patch). Stratification by previous NRT patch use indicated that the estimated varenicline treatment effect versus NRT was numerically greater in individuals who had previously used NRT, although the interaction tests were not significant (p = 0.155 for end of treatment CAR and p = 0.681 through week 52). However, previous use of NRT did not account for the between-country differences in the relative efficacy of varenicline at the end of treatment or through week 52.

**Table 2:** Continuous abstinence rate by country over last 4 weeks of treatment

Last 4 weeks of treatment	Country				
	Belgium	France	The Netherlands	UK	USA
Varenicline (weeks 9–12), n/N (%)	26/55 (47.3)	18/35 (51.4)	39/65 (60.0)	67/116 (57.8)	60/105 (57.1)
NRT (weeks 8–11), n/N (%)	31/53 (58.5)	11/31 (35.5)	38/64 (59.4)	35/115 (30.4)	45/107 (42.1)

NRT, nicotine replacement therapy.

#### 7-day point prevalence of abstinence

The 7-day point prevalence of abstinence score at week 12 in the Primary Analysis Population was significantly higher for varenicline (62.0%) than for NRT (47.0%; OR 1.71, 95% CI 1.27 to 2.30, p<0.001). There were no significant differences at week 24 (varenicline, 38.6%; NRT, 34.1%; OR 1.22, 95% CI 0.90 to 1.66, p = 0.193) or at week 52 (varenicline, 34.8%; NRT, 31.4%; OR 1.18, 95% CI 0.87 to 1.62, p = 0.285).

#### Craving, withdrawal symptoms and reinforcement

Scores in the urge to smoke subscale of the MNWS were lower for varenicline than for NRT at each weekly time point. When these data were averaged over weeks 1–7, varenicline scores were significantly lower than NRT scores (p<0.001), indicating that varenicline reduced craving further than NRT. Varenicline also showed significantly lower scores in the negative effect (p<0.001) and restlessness (p<0.001) subscales of the MNWS, but not the increased appetite or insomnia subscales (table 3).

**Table 3:** Measures of withdrawal and craving using the Minnesota Nicotine Withdrawal Scale (MNWS): repeated measures analysis of data for weeks 1–7

	N	LS Mean (SE)*	Varenicline vs NRT			
			Difference (SE)	95% CI	p Value	Effect size†
Varenicline						
Urge to smoke	367	1.35 (0.04)	−0.32 (0.06)	–0.44 to −0.21	<0.001	–0.37
Negative effect	369	0.63 (0.03)	–0.16 (0.04)	–0.24 to –0.07	<0.001	–0.21
Restlessness	368	0.76 (0.04)	–0.20 (0.05)	–0.31 to –0.10	<0.001	–0.21
Increased appetite	368	1.07 (0.04)	0.09 (0.06)	–0.02 to 0.21	0.116	0.12
Insomnia	368	0.70 (0.04)	−0.07 (0.05)	–0.17 to 0.04	0.207	−0.07
						
NRT						
Urge to smoke	366	1.67 (0.04)				
Negative effect	366	0.79 (0.03)				
Restlessness	366	0.96 (0.04)				
Increased appetite	364	0.97 (0.04)				
Insomnia	366	0.76 (0.04)				

NRT, nicotine replacement therapy.

*Higher scores indicate greater intensity of symptoms. Response scale: 0 (not at all) to 4 (extreme).

†Effect size, LS mean difference divided by the pooled SD at baseline (pooled by centre).

Compared with NRT, varenicline participants reported significantly lower smoking satisfaction (p<0.001), psychological reward (p = 0.001), enjoyment of respiratory tract sensations (p<0.001) and craving reduction (p<0.001) in the average week 1–7 scores of the mCEQ. There was no significant difference between the aversion subscale scores for the two groups (table 4).

**Table 4:** Measurement of smoking reinforcement using the modified Cigarette Evaluation Questionnaire (mCEQ): repeated measures analysis of data for weeks 1–7 only in those who had smoked since their last visit

	N	LS Mean (SE)*	Varenicline vs NRT			
			Difference (SE)	95% CI	p Value	Effect size†
Varenicline						
Smoking satisfaction	361	2.73 (0.09)	–0.54 (0.12)	–0.77 to –0.31	<0.001	–0.43
Psychological reward	361	2.30 (0.07)	–0.32 (0.10)	–0.51 to –0.13	0.001	–0.26
Enjoyment of respiratory tract sensations	358	2.04 (0.08)	–0.39 (0.11)	–0.60 to –0.17	<0.001	–0.25
Craving reduction	360	3.62 (0.10)	–0.52 (0.14)	–0.79 to –0.24	<0.001	–0.32
Aversion	361	1.76 (0.07)	–0.07 (0.09)	–0.25 to 0.11	0.436	–0.08
						
NRT						
Smoking satisfaction	354	3.27 (0.08)				
Psychological reward	354	2.61 (0.07)				
Enjoyment of respiratory tract sensations	353	2.42 (0.08)				
Craving reduction	354	4.14 (0.10)				
Aversion	354	1.83 (0.06)				

NRT, nicotine replacement therapy.

N, total number of participants contributing to the repeated measures analysis. To be included subjects must have completed a questionnaire at baseline and at one or more post-baseline visits. This includes those who smoked during the first week of treatment, which was before the target quit date.

*Higher scores indicate greater intensity of smoking effects. Response scale: 1 (not at all) to 7 (extreme).

†Effect size, LS mean difference divided by the pooled SD at baseline (pooled by centre).

### Safety

Overall, treatment-emergent adverse events were experienced by 84.8% of the varenicline group and 70.3% of the NRT group (table 5). Most adverse events were reported as mild or moderate in intensity. The all-cause treatment-emergent adverse events are summarised in table 5. The most frequent adverse events in both treatment groups were nausea, insomnia, headache and abnormal dreams. Severe adverse events were experienced by 9.8% of the varenicline group and 7.3% of the NRT group. The most commonly reported severe adverse events were nausea (varenicline, n = 7; NRT, n = 0), headache (varenicline, n = 5; NRT, n = 0) and insomnia (varenicline, n = 5; NRT, n = 1). 

**Table 5:** Summary of all-cause treatment-emergent adverse events occurring in ⩾5% of either treatment group

Parameter	Varenicline 1 mg twice daily(n = 376)	Nicotine titrated from 21 mg patches(n = 370)
Any adverse event	319 (84.8)	260 (70.3)
Treatment discontinuations due to adverse events	30 (8.0)	16 (4.3)
Dose reductions or temporary withdrawal from study medication	44 (11.7)	25 (6.8)
Deaths	0 (0.0)	0 (0.0)
Serious adverse events	2 (0.5)	8 (2.2)
Most frequent adverse events		
Nausea	140 (37.2)	36 (9.7)
Insomnia	80 (21.3)	71 (19.2)
Headache	72 (19.1)	36 (9.7)
Abnormal dreams	44 (11.7)	31 (8.4)
Constipation	31 (8.2)	9 (2.4)
Dizziness	28 (7.4)	13 (3.5)
Disturbance in attention	24 (6.4)	5 (1.4)
Vomiting	23 (6.1)	4 (1.1)
Diarrhoea	22 (5.9)	10 (2.7)
Flatulence	22 (5.9)	5 (1.4)
Dysgeusia	22 (5.9)	4 (1.1)
Abdominal pain (upper)	21 (5.6)	4 (1.1)
Fatigue	21 (5.6)	9 (2.4)

Data presented as n (%).

Table 5 shows permanent discontinuations from study medications due to adverse events and temporary withdrawal from or reduction in doses of study medication due to treatment-emergent adverse events. The most frequent adverse event leading to treatment discontinuation was nausea (varenicline, 2.1%; NRT, 0.8%). No other adverse event resulted in treatment discontinuation in >1% of the population.

Ten participants experienced serious adverse events while receiving treatment. One participant in the varenicline group had depression that was attributed to the study drug. Treatment was discontinued. Another participant in the varenicline group had constipation that was not considered related to study medication. The remaining eight participants were in the NRT group and experienced bile duct cancer and sepsis; gastrointestinal bleeding; myocardial infarction (n = 2); salivary gland tumour; chest pain (n = 2); and worsening of existing knee trauma. None of these serious adverse events was attributed to NRT.

Three participants experienced serious adverse events during the non-treatment follow-up phase. In the varenicline group, one participant experienced acute ethanol intoxication. A woman in the varenicline group experienced suicidal ideation which resulted in hospitalisation 11 days after completing the varenicline treatment. She had stopped smoking and was experiencing family turmoil; she had a history of moodiness, but no diagnosed disorder. The study investigator considered this case to be attributable to the study drug. In the NRT group, one participant experienced an abdominal cyst that was not considered to be related to NRT. No participants died during the study.

There were no permanent discontinuations of study medication as a result of laboratory test abnormalities and no clinically relevant differences were shown between treatment groups for any laboratory test. One participant in the varenicline group had a raised liver function test reading at the end of treatment but was then lost to follow-up so no additional testing was conducted.

The mean change in body weight from baseline to the end of treatment for all participants (regardless of smoking status) was 1.82 kg for varenicline (week 12, n = 262) and 1.62 kg for NRT (week 11, n = 229). The mean change in body weight for participants who remained continuously abstinent for the last 4 weeks of treatment was 2.02 kg (n = 188) for varenicline and 2.07 kg (n = 131) for NRT.

## DISCUSSION

In this open-label study, smokers motivated to quit had significantly higher abstinence rates during the last 4 weeks of treatment when treated with 12 weeks of varenicline than with 10 weeks of transdermal NRT. The CAR for both treatment groups was similar for the pre-specified Primary Analysis Population and the All Randomised Population and the superior treatment effect of varenicline was also evident with similar effect sizes at week 11 (end of NRT treatment) and at week 12 (end of varenicline treatment). The CAR difference did not remain significant through week 52 (NRT, weeks 8–52; varenicline, weeks 9–52) in the Primary Analysis Population. The 7-day point prevalence of abstinence rates were similar to the CAR outcomes.

Many researchers consider an intent-to-treat approach, where the analysis population includes all randomised participants regardless of whether they actually received study drug treatment, as the gold standard for reporting most clinical trials. However, we report the Primary Analysis Population (those who were randomised *and* took at least one dose of medication) in the efficacy results as this was our prespecified primary analysis population. These results may underestimate the efficacy of varenicline relative to NRT because of differential dropout after medication assignment (ie, more participants withdrew from the study after randomisation and before receiving a first dose of medication in the NRT group (n = 9; 2%) than in the varenicline group (n = 2; 0.5%)). Since this was an open-label study, the differential dropout may have been due to dissatisfaction with treatment assignment. Consequently, it is important to include the results of the prespecified analyses for both populations. The All Randomised analyses statistically favour varenicline over NRT at the end of treatment CAR and week 52 CAR (NRT, weeks 8–52; varenicline, weeks 9–52).

The odds of abstinence at the end of open-label treatment for varenicline over NRT (OR 1.70) is the same as that reported for abstinence 4 weeks after the quit date in the comparison of varenicline versus NRT in consecutive routine cases before and after the introduction of varenicline.^13^ This also approximates recent varenicline versus bupropion SR double-blind comparisons showing ORs of 1.90 and 1.93 at the end of treatment.^8^ ^10^ In the longer term (over 52 weeks) the OR of remaining abstinent with open-label varenicline versus NRT in this study was 1.40, which is similar to the OR for double-blind varenicline versus bupropion SR through week 52 in one previous study (1.46)^8^ and lower than in another study (1.77).^10^ In a meta-analysis of trials comparing transdermal NRT to placebo or no patch, the estimated long-term abstinence rate (after 52 weeks from the start of treatment with transdermal NRT) was 13.7%,^6^ compared with the CAR of 20.3% through week 52 in the present study. The reasons for the higher abstinence rates in this study are unclear.

In a recent meta-analysis using indirect comparisons of treatment effect versus placebos, varenicline was found to be significantly superior to NRT at 3 months (OR 1.78) and at 12 months (OR 1.66) while bupropion SR versus NRT at 12 months showed no difference (OR 1.14). The direct comparisons of varenicline versus NRT reported here are similar to these indirect comparisons but with a lower difference at 12 months.^20^

Preclinical studies have shown that varenicline acts as a partial agonist at the α4β2 nicotinic acetylcholine receptor (nAChR), with both agonist and antagonist effects.^21^ ^22^ Participants who continued to smoke in the varenicline group reported a greater reduction in smoking satisfaction than continuing smokers in the NRT group. This may reflect the antagonist action of varenicline at α4β2 nAChRs which are thought to mediate the reinforcing effects of nicotine. As an agonist, nicotine would be expected to alleviate withdrawal symptoms. In this study, varenicline showed significant benefit over NRT in measures of craving and withdrawal by decreasing the urge to smoke, negative effect and restlessness. These findings are consistent with lower severity ratings of craving in participants in a routine smoking cessation clinic taking varenicline compared with those taking NRT,^13^ and are encouraging in terms of increasing the pharmacotherapy choices for smokers who find it hard to cope with symptoms of withdrawal during quit attempts. This benefit of a partial agonist over a full agonist is perhaps surprising, but may be due to the combined effect of nicotine concentrations from NRT being lower than those obtained from regular smoking and varenicline having a higher affinity than nicotine for the α4β2 nAChR.

Safety data for both treatment groups were consistent with previous reports,^23^ ^24^ although the occurrence of adverse skin reactions in the transdermal nicotine group in this study was relatively low and may have been due to the exclusion of participants with allergic reactions to adhesive tape and skin disorders. The overall number of treatment discontinuations due to adverse events in this study (8%) was similar to the number reported in previous varenicline versus bupropion SR comparator trials (8.6%^8^ and 10.5%^10^) and lower than the numbers reported for bupropion SR (15.2%^8^ and 12.6%^10^). Reports of nausea (37.2%), insomnia (21.3%), headache (19.1%) and constipation (8.2%) were more frequent in this trial than those previously reported. The outcomes of a pooled analysis of the frequency of adverse events occurring in the two phase 3 comparator trials of varenicline versus bupropion SR were: nausea (28.8% varenicline vs 9.9% bupropion SR); insomnia (14.2% varenicline vs 21.5% bupropion SR); headache (14.2% varenicline vs 11.1% bupropion SR); and constipation (7.2% varenicline v 6.7% bupropion SR).^25^ Some physiological side effects reported by participants may be due to smoking cessation rather than treatment medication. For example, insomnia is a DSM IV (*Diagnostic and Statistical Manual of Mental Disorders*, 4th edition) nicotine withdrawal symptom^26^ and constipation has also been proposed as an additional symptom of withdrawal from nicotine.^27^  Weight gain is also listed as a withdrawal symptom in DSM IV.^26^ The mean weight gain for participants in both treatment groups at the end of treatment was similar, regardless of smoking status.

### Limitations of the study

A limitation of this study was the open-label design. The differential dropout rate after medication assignment and before the first dose of treatment suggests that some motivational bias may have influenced the results. Discontinuation for adverse events was less likely with NRT than with varenicline, while a refusal to participate further was less likely with varenicline than with NRT. A double-blind design may have avoided such biases. A truly blind comparison of varenicline tablets and NRT patch would have required a double-dummy design using identical active drug and placebo tablets and patches. However, technical problems made it difficult to create NRT and placebo patches that were indistinguishable from one another in appearance and odour. Motivational influences are likely to exist in a real-world setting and the outcomes of this study show that varenicline is more effective than transdermal nicotine in enhancing quit rates in an open-label setting.^13^

There was a longer treatment duration with varenicline (12 weeks) than with NRT (10 weeks), in accordance with the prescribing guidelines for each product,^14^ ^15^ which may have led to a treatment bias. However, a recent meta-analysis found no benefit of prolonged transdermal NRT treatment beyond 8 weeks,^6^ and the effect sizes of varenicline versus transdermal NRT were similar in weeks 8–11 and weeks 9–12 of this study.

Missing CO measurements were imputed as ⩽10 ppm so, if all other criteria for abstinence were met, missing CO confirmation did not change the status of participants to smoker. However, this did not have any meaningful impact on the results. An alternative method for imputing missing CO measurements that is recommended by the Russell Standards for analysis of smoking studies^28^ is to impute the missing CO as negative (not smoking for the CAR end points) if the subsequent measurement is not positive and at least the last measurement (ie, the week 52 measurement) is non-missing and negative. If we apply this imputation method, the result is one fewer NRT non-smoker in the end of treatment CAR and one fewer varenicline non-smoker for the week 52 CAR (NRT, weeks 8–52; varenicline, weeks 9–52).

Subject selection criteria may also limit the interpretation of the results.^29^ The superiority of varenicline over NRT may not generalise to populations such as teenage smokers, medically compromised smokers (eg, those with active cardiac problems) or smokers with current psychiatric disorders. There were very few non-Caucasian participants in this study, although recent studies conducted in Taiwan, Korea and Japan have shown that varenicline has significant efficacy over placebo in these populations.^30^ ^31^

With regard to future directions for research, trials are currently being conducted in participants with cardiovascular disease (www.clinicaltrials.gov; identifier: NCT00282984) and chronic obstructive pulmonary disease (www.clinicaltrials.gov; identifier: NCT00285012). There is some limited evidence that varenicline is effective and safe in adults receiving treatment for mental illness.^13^ However, given the strict exclusion criteria for phase 3 trials, future studies should also evaluate the efficacy of varenicline in other specific populations such as those with psychiatric disorders and adolescent smokers.

In conclusion, in this open-label comparison of varenicline with transdermal NRT, varenicline appeared to be safe and well tolerated. Varenicline demonstrated significantly greater abstinence rates than NRT at the end of treatment, consistent with previously reported trials of varenicline versus placebo and bupropion SR.^8^ ^10^ In addition, varenicline showed a greater reduction on measures of craving, withdrawal and feelings of smoking satisfaction compared with NRT.
